# Supplementary material for: Parental Effect on Agronomic and Olive Oil Traits in Olive Progenies from Reciprocal Crosses
Source: Plants (Basel). 2024 Sep 3;13(17):2467. doi: 10.3390/plants13172467 (PMC11396948; doi:10.3390/plants13172467)
Supplement: Supplementary file 1 [file plants-13-02467-s001.zip › plants-3168667-supplementary.pdf]

Table S1. Descriptive statistical analysis results of each reciprocal cross ('Arbosana' × 'Sikitita' and 'Sikitita' × 'Arbosana') and of the reference cultivars 'Arbosana' and 'Arbequina' on the agronomic traits (A), phenolic compounds (B), fatty acids and sterols (C) of virgin olive oil. The results show minimum, maximum, median, mean, SD (standard deviation) and CV (coefficients of variation) values for each of the evaluated traits.

A. Agronomic traits

**Statistical index**	**Ripening index**	**Tree height (cm)**	**Tree Width (cm)**	**Trunk diameter (mm)**	**Average fruit weight (g)**	**Average endocarp weight (g)**	**Flesh / Stone ratio**	**Fresh matter oil content (%)**	**Dry matter oil content (%)**	**Fruit moisture (%)**	**Oxidative stability (h)**
‘Arbosana’ × ‘Sikitita’											
**Minimum**	0.00	180.00	210.00	61.00	0.78	0.14	72.86	10.20	29.12	55.84	26.60
**Maximum**	3.00	410.00	340.00	108.00	2.56	0.49	85.86	20.70	54.20	65.72	126.70
**Median**	1.00	315.00	285.00	79.50	1.57	0.32	80.36	15.90	40.54	61.02	46.00
**Mean**	1.31	312.50	272.50	80.94	1.61	0.32	79.76	15.84	40.55	61.07	54.08
**SD**	0.66	59.27	40.25	13.41	0.55	0.09	3.91	2.76	5.76	2.67	26.28
**CV**	0.50	0.19	0.15	0.17	0.34	0.29	0.05	0.17	0.14	0.04	0.49
‘Sikitita’ × ‘Arbosana’											
**Minimum**	0.50	220.00	200.00	62.00	0.87	0.20	67.33	11.35	32.60	51.81	23.90
**Maximum**	4.00	380.00	310.00	120.00	2.66	0.49	88.22	24.89	56.91	69.89	121.30
**Median**	1.50	300.00	250.00	81.00	1.70	0.29	83.21	16.25	43.67	61.55	52.50
**Mean**	1.74	302.94	258.82	82.18	1.75	0.30	82.06	16.63	42.67	61.12	59.17
**SD**	0.92	41.35	32.96	14.29	0.48	0.07	5.57	3.61	6.49	4.79	28.44
**CV**	0.53	0.14	0.13	0.17	0.27	0.24	0.07	0.22	0.15	0.08	0.48
‘Arbosana’											
**Minimum**	0.50	260.00	210.00	59.00	1.04	0.16	80.85	13.75	38.10	58.70	39.20
**Maximum**	1.00	330.00	300.00	81.00	1.55	0.25	85.71	18.39	45.75	66.27	57.20
**Median**	1.00	315.00	250.00	70.00	1.22	0.19	84.59	15.27	43.92	64.53	48.80
**Mean**	0.87	305.00	252.50	70.00	1.26	0.20	83.93	15.67	42.92	63.50	48.50
**SD**	0.25	33.16	37.74	9.01	0.23	0.04	2.15	2.02	3.36	3.34	7.59
**CV**	0.29	0.11	0.15	0.13	0.18	0.24	0.03	0.13	0.08	0.05	0.16
‘Arbequina’											

**Minimum**	1.50	300.00	270.00	64.00	1.10	0.18	80.13	12.77	36.43	62.36	26.20
**Maximum**	2.00	390.00	360.00	96.00	1.69	0.31	85.49	14.53	42.48	65.80	42.60
**Median**	1.50	365.00	315.00	82.50	1.32	0.24	81.72	13.89	37.85	64.59	37.85
**Mean**	1.63	355.00	315.00	81.25	1.36	0.24	82.26	13.77	38.65	64.33	36.13
**SD**	0.25	40.42	36.97	15.69	0.26	0.06	2.28	0.75	2.79	1.54	7.18
**CV**	0.15	0.11	0.12	0.19	0.19	0.24	0.03	0.05	0.07	0.02	0.20

B. Phenolic profile

				'Arbosana' × 'Sikitita'					
**Statistical index**	**Hydroxytyrosol (mg/kg)**	**Tyrosol (mg/kg)**	**Oleacein (mg/kg)**	**Oleocanthal (mg/kg)**	**Oleuropein aglycone (mg/kg)**	**Ligstroside Aglycone (mg/kg)**	**Apigenin (mg/kg)**	**Luteolin (mg/kg)**	**Total (mg/kg)**
Minimum	0.00	1.40	1.50	1.30	1.70	0.00	0.00	0.70	13.50
Maximum	5.00	15.60	262.00	426.00	95.30	48.60	9.20	11.10	649.50
Median	0.60	3.80	80.75	52.90	32.60	12.30	3.60	2.00	180.50
Mean	0.88	5.13	79.26	70.67	38.58	14.75	3.59	3.40	216.26
SD	1.09	3.58	65.21	90.78	29.74	13.45	2.85	2.69	150.71
CV	1.24	0.70	0.82	1.28	0.77	0.91	0.79	0.79	0.70
				'Sikitita' × 'Arbosana'					
Minimum	0.00	2.00	4.50	6.40	7.40	1.20	0.00	0.90	27.80
Maximum	6.90	14.00	300.00	182.00	166.00	77.70	7.80	9.70	619.70
Median	1.10	5.90	92.80	60.15	25.80	11.55	1.20	2.55	203.90
Mean	1.82	6.50	100.24	70.55	47.52	18.87	2.18	2.83	250.51
SD	1.98	3.34	84.70	56.95	45.28	19.46	2.36	2.07	163.76
CV	1.09	0.51	0.85	0.81	0.95	1.03	1.08	0.73	0.65
				'Arbosana'					
Minimum	0.00	2.70	57.70	59.60	14.70	3.50	2.80	0.30	149.20
Maximum	0.40	3.80	197.00	169.00	31.40	12.40	4.90	3.30	417.80
Median	0.05	3.00	112.00	83.45	20.50	5.40	3.95	1.90	229.85

Mean	0.11	3.09	114.74	98.46	21.40	6.69	3.81	1.81	250.11
SD	0.16	0.37	44.44	39.93	5.52	3.48	0.72	1.08	90.26
CV	1.38	0.12	0.39	0,41	0.26	0.52	0.19	0.60	0.36
					‘Arbequina’				
Minimum	0.00	1.90	30.40	18.30	6.90	0.00	0.00	1.70	62.10
Maximum	2.50	6.20	154.00	54.60	21.00	3.10	3.40	3.90	230.60
Median	0.15	2.95	79.65	48.40	15.15	0.10	1.25	2.95	156.55
Mean	0.55	3.40	86.24	41.70	13.94	0.79	1.33	2.99	150.93
SD	0.84	1.52	45.50	14.81	4.80	1.15	0.98	0.75	61.28
CV	1.52	0.45	0.53	0.36	0.34	1.46	0.74	0.25	0.41

C. Fatty acids and Sterols profile						
			‘Arbosana’ × ‘Sikitita’			
**Statistical index**	**SFA%**	**MUFA%**	**PUFA%**	**Campesterol %**	**β-sitosterol %**	**Δ5-avenasterol %**
Minimum	12.51	67.45	2.09	3.15	83.04	2.30
Maximum	21.31	85.00	12.64	4.50	90.71	10.36
Median	18.48	72.92	8.96	3.93	88.46	5.49
Mean	17.61	74.12	8.26	3.85	87.81	5.45
SD	2.46	5.49	3.39	0.44	2.48	2.58
CV	0.14	0.07	0.41	0.11	0.03	0.47
			‘Sikitita’ × ‘Arbosana’			
Minimum	14.49	53.20	2.23	2.45	81.24	2.64
Maximum	27.61	82.78	26.16	4.70	90.49	12.58
Median	17.50	75.77	8.89	3.75	86.33	7.31
Mean	18.17	72.23	9.60	3.67	86.25	7.08
SD	3.32	9.50	6.88	0.59	2.83	3.20

CV	0.18	0.13	0.72	0.16	0.03	0.45
			'Arbosana'			
Minimum	17.55	73.35	7.33	3.47	83.56	7.79
Maximum	18.41	74.77	8.60	3.64	85.50	9.76
Median	17.95	73.73	8.25	3.54	84.73	8.32
Mean	17.97	73.90	8.14	3.55	84.63	8.55
SD	0.31	0.56	0.49	0.07	0.83	0.93
CV	0.02	0.01	0.06	0.02	0.01	0.11
			'Arbequina'			
Minimum	17.68	63.67	12.74	3.49	84.64	5.67
Maximum	20.17	69.57	16.20	4.40	86.49	8.95
Median	18.68	66.55	14.61	3.60	85.49	7.92
Mean	18.83	66.54	14.63	3.77	85.53	7.61
SD	0.96	2.29	1.35	0.43	1.03	1.62
CV	0.05	0.03	0.09	0.11	0.01	0.21
